# Transfer of cGAMP from neuron to microglia activates microglial type I interferon responses after subarachnoid hemorrhage

**DOI:** 10.1186/s12964-023-01362-3

**Published:** 2024-01-02

**Authors:** Hanxiao Chang, Zheng Li, Weiwei Zhang, Chao Lin, Yuqi Shen, Guangjian Zhang, Lei Mao, Chencheng Ma, Ning Liu, Hua Lu

**Affiliations:** 1 Department of Neurosurgery, Jiangsu Province Hospital and Nanjing Medical University First Affiliated Hospital, Nanjing, 210029 Jiangsu China; 2 Department of Ophthalmology, Third Medical Center of Chinese, PLA General Hospital, Beijing, 100000 China

**Keywords:** cGAS, mtDNA, cGAMP, Interferon reaction, SAH

## Abstract

Primary subarachnoid hemorrhage (SAH) is a type of acute stroke, accounting for approximately 10% of cases, with high disability and mortality rate. Early brain injury (EBI) is a critical factor in determining SAH mortality; however, there are no effective treatment interventions for EBI. Based on our results, the transmission of cyclic GMP-AMP (cGAMP) from neurons to microglia is a key molecular event that triggers type I interferon response, amplifies neuroinflammation, and leads to neuronal apoptosis. Abnormal intracytoplasmic mitochondrial DNA (mtDNA) is the initiating factor of the cGAS-cGAMP-STING signaling axis. Overall, the cGAS-cGAMP-STING signaling axis is closely associated with neuroinflammation after subarachnoid hemorrhage. Targeting cGAS triggered by cytoplasmic mtDNA may be useful for comprehensive clinical treatment of patients after SAH. Further studies targeting cGAS-specific antagonists for treating SAH are warranted.

Video Abstract

**Supplementary Information:**

The online version contains supplementary material available at 10.1186/s12964-023-01362-3.

## Introduction

Primary subarachnoid hemorrhage (SAH) is a type of acute stroke, accounting for approximately 10% of cases, with a high disability and mortality rate. In the past few decades, researchers have investigated the pathological mechanisms that contribute to SAH, including early brain injury (EBI) and delayed cerebral ischemia (DCI) [1–3]. Early brain injury is a critical determining factor for SAH mortality; however, there is no effective treatment intervention for it [1]. Recently, extensive studies suggest that excessive neuroinflammation after SAH is associated with neurological deficits. Anti-neuroinflammatory treatment has become a focus of clinical and basic scientific research [4, 5].

The pathogenesis of several acute conditions (stroke and traumatic brain injury) and chronic neurodegenerative diseases (Parkinson’s disease and Alzheimer’s disease) is associated with neuroinflammation [6, 7]. Activated microglia, particularly M1-type microglia, play a crucial role in the development of post-SAH neuroinflammation. Promoting the conversion of microglia phenotype from pro-inflammatory M1 to anti-inflammatory M2 form might be beneficial to post-SAH early brain injury. Moreover, previous studies have shown that inhibiting neuroinflammation has a neuroprotective effect in post-SAH early brain injury [7]. Neuroinflammation after SAH is usually activated by endogenous substances and type I interferon response caused by cytosolic double-stranded DNA may be involved in the activation of neuroinflammation.

The cGAS-STING pathway, involving the synthase for the second messenger cyclic GMP-AMP (cGAS) and the cyclic GMP-AMP receptor stimulator of interferon genes (STING), detects endogenous and exogenous double-stranded DNA to trigger an innate immune reaction involving a strong type I interferon response against infections and stress damage [8, 9]. In addition to exogenous DNA from microorganisms and endogenous nuclear DNA, the type I interferon response is also activated by cytosolic mtDNA.

Mitochondria, commonly referred to as the powerhouse of the cell, are the sites of oxidative phosphorylation and energy production, which occur in the form of ATP. In recent years, mitochondrial dysfunction after subarachnoid hemorrhage has been gradually recognized as a key factor affecting clinical prognosis. Meanwhile, recent studies have shown that mitochondrial double-stranded DNA released into the cytoplasm after mitochondrial injury can activate the cGAS-STING pathway and induce a type I interferon response, aggravating the inflammatory response of the nervous system [10–13].

In this study, we investigated the proinflammatory role of the mtDNA-cGAS-STING pathway following SAH. We demonstrated that neuron-derived cGAMP promotes microglial polarization to the M1 type and triggers excessive neuroinflammation.

## Experimental design

### SAH model

We purchased twelve-weeks old male C57BL/6 mice weighing 22–26 g from Charles River (Beijing, China) and maintained them in a specific pathogen free (SPF) conditions. All experimental procedures were performed in accordance with the approved guidelines and protocols of the Nanjing Medical University (NMU). The Animal Care and Use Committee of the NMU approved all the animal experiments. The ethical approval reference number is IACUC 2005019.

Animal grouping and neurological testing were blinded, and the inclusion and exclusion criteria were determined by the animals’ health status after the experiment. Mice that died from nonsurgical causes were replenished.

We used the endovascular perforation model to induce SAH in mice as previously described [14]. We briefly exposed the mice to pentobarbital anesthesia (5 mg/10 g), and gently pushed the filament forward from the isolated external carotid artery stump. After feeling resistance, we pushed the filament by an additional 1 mm to puncture the vessel. Sham mice underwent the same procedure without puncturing. Mice were killed by cervical dislocation, and efforts were made to minimize animal suffering.

H-151 treated mice were injected intraperitoneally with 750 nmol H151 (941987–60–6, Invivogen, USA) every 24h, beginning at 72h before surgery.

### Cell culture

The dissociated glial cells of C57BL/6 mice were isolated from whole brain tissues from one or two postnatal days.

After dissecting the skull and perichondrium in a 4 °C pre-cooled buffer (10% horse serum and 2% penicillin and streptomycin in DMEM:F12 (1:1)), the brain tissue was dissected and digested with 0.25% trypsin at 37 °C for 30 min, then filtered through a 100-μm strainer to eliminate tissue fragments. The resuspended cells were transferred to d-lysine-coated culture flasks. The glial cell medium was a high-glucose medium (DMEM, Gibco, USA) containing 10% fetal calf serum, 100 U/ml penicillin, and 100 mg/ml streptomycin (Gibco, USA). glial cells formed a confluent monolayer. Microglia were separated from astrocytes by shaking the flask and were collected by centrifuging. We separated microglia from astrocytes by shaking the flask and collected them by centrifugation after 14 days of seeding. 

We obtained primary neurons from the cerebral cortex of 17-days old embryonic C57BL/6 mice. After dissecting the skull and perichondrium in a 4 °C pre-cooled buffer (10% horse serum and 2% penicillin and streptomycin in DMEM:F12 (1:1)), the brain tissue was dissected and digested with 0.25% trypsin at 37 °C for 30 min. Then we filtered and eliminated tissue fragments by 70μm strainer. After pelleting by centrifugation at 200 × g for 5 min, we obtained the cell mass containing neurons and glial cells. The cell mass were resuspended with DMEM: F12 (1:1) supplemented with 10% horse serum and 2% penicillin and streptomycin (Gibco) and cultured them in twelve-well plate (3.0 × 10^5^ cells per cell) coated with D-lysine. At about 5 h after seeding, we removed the primary culture solution. At this point, the neurons were already adherent and the glial cells were removed. After seeding with neurobasal containing 1% B27 and 0.5 mM glutamate for 7 days, the primary neurons were used for the experiment.

Concerning the neuron and microglia co-culture system, microglia were seeded in Transwell (Corning, pore size = 0.4 μm) upper chamber and the neurons were seeded in the plates. The co-culture medium was DMEM with 10% fetal bovine serum (FBS).

We blocked the Lrrc8/VRAC channels by endovion and the mPTP opening in neurons with 10 μM cyclosporine (CsA).

### Neurological severity score

We evaluated neurological function with the scoring system previously described by Garcia et al. Briefly, the following clinical conditions were assessed (score range 0–3 or 1–3): spontaneous activity, symmetry in the movement of four limbs, forepaw outstretching, climbing, body proprioception, and response to vibrissae touch. A low score indicated severe neurological damage [14].

### TUNEL assay

We measured apoptotic cell death using the TUNEL assay (One Step TUNEL Apoptosis Assay Kit, Beyotime, China, No.C1089). Cryosections (20 μm thick) were incubated with 50 μL of the TUNEL reaction mixture. Apoptosis was calculated as TUNEL-positive cells (red)/ DAPI-stained nuclei (blue).

### Immunohistochemistry assay

We performed immunohistochemistry assays using a streptavidin–biotin immunoperoxidase assay. Briefly, the 20 μm thick cryosections were incubated primary antibodies for 8h at 4 °C, secondary antibody for 30 min at ambient temperature, and DAB for 15 min at 37 °C successively.

### Immunofluorescence staining

The 20 μm thick cryosections and neurons were fixed in 4% paraformaldehyde for 30 min at 25°C and then blocked with 5% goat serum and 0.2% Triton X-100 diluted in PBS for 1 h at 25°C. After that, the 20 μm thick cryosections and neurons were incubated with primary antibodies for 8h at 4°C, secondary antibody for 30 min at ambient temperature, and DAPI for 5 min at 37 °C successively.

### PI assay

The neurons were incubated with Calcein and PI mixed liquor (Beyotime, C2015, Shanghai, China) for 30min at 37℃. The cells were imaged and counted under an inverted fluorescence microscope.

### Nissl stain

The 20 μm thick cryosections were incubated with Nissl staining solution (Beyotime, C0117, Shanghai, China) for 5 min at 37 °C and washed with 95% ethyl alcohol for 5 min, and xylene for 5 min successively.

### Mito-track and mitoSOX imaging

Mito-track imaging: After washing with PBS, the neurons were incubated with PBS containing 200 nM MitoTracker Deep Red FM (Invitrogen) for 30 min.

MitoSOX imaging: After washing with PBS, the neurons were incubated with PBS containing 5 μM red mitochondrial superoxide indicator (Invitrogen) for 10 min.

### Transmission electron microscopy

After fixation with 2.5% glutaraldehyde, the mice brain samples were post-fixed with 1.5% osmium tetroxide for 2h at 4°C, dehydrated with ethanol, and embedded in epoxy resin. The ultrastructure of the samples (100- nm ultrathin sections) was observed using a transmission electron microscope (Quanta 10, FEI Co.).

### IL-1β, IL-6, TNF-α, CXCL-10 and cGAMP levels

Interleukin-6, IL-1β, TNF-α, CXCL-10, and cGAMP levels were assessed using a specifc enzyme-linked immunosorbent assay according to the manufacturer’s instructions. IL-1β (Beyotime, P1301, Shanghai, China), IL-6 (Beyotime, PI326, Shanghai, China), TNF-α (Beyotime, PT512, Shanghai, China), CXCL-10 (Beyotime, PC206, Shanghai, China), and cGAMP(Meimian, 44862M2, China).

### Western blot analysis

After extraction using a cytosol and cell membrane protein extraction kit (Beyotime, P0033, China), the proteins were separated using SDS-PAGE and transferred to polyvinylidene fluoride (PVDF) membrane. The PVDF membranes were blocked with bovine serum albumin (5%), incubated with a primary antibody for 8 h at 4°C, and incubated with a secondary antibody at 25°C. Finally, we visualized the PVDF membranes using the SuperSignal Maximum Sensitivity Substrate (Thermo Fisher Scientific).

### Cell transfection

We transfected siRNA into neurons and microglia using Lipofectamine 2000 transfection reagent (Invitrogen, Carlsbad, CA, USA) in accordance with the manufacturer's protocol, and evaluated transfection effectiveness using western blotting. We purchased si-cGAS and si-STING from GenePharma (Shanghai, China).

### Flow cytometry

We tested the mitochondrial membrane potential using JC-1 Assay Kit (Beyotime, China) according to the manufacturer’s instructions and measured cell fluorescence using flow cytometry.

The single primary microglia suspension was counted and stained with anti-mouse CD11b-FITC (Proteintech, China, No.65055) and CD86-APC (Proteintech, China, No.65068), and cell fluorescence was measured by flow cytometry.

### Brain water content

To determine the wet weight, we removed and weighed the mouse brains after sacrifice. Subsequently, we determined the dry weight after drying at 70°C for 72 h. Finally, the brain water content was determined using the formula:

Water content (%) = [(wet weight-dry weight)/wet weight] × 100.

### Morris water maze (MWM)

The Morris water maze (MWM) was performed by the same experimenter at the same time between 08:30 and 12:00 each day to assess spatial learning and memory. In each experiment, the mice were placed at a random starting point. If the mice failed to reach the platform within the allotted time, they were selected and placed on the platform for 15 s. In the probe test, the mice were placed in a tank on the opposite side of the target quadrant and looked for a platform for 60 s. The hidden platform test was conducted on days 1–5 of the test. On the fifth day, the probe test was performed 2 h after the hidden platform test. Both visible platform tests were conducted on days 6 or 7 of the test. Latency, distance traveled, and number of entries were analyzed using a tracking device and software (Chromotrack 3.0, San Diego Instruments).

### dsDNA extraction in cytosol

We removed mitochondria and isolated the cytosolic fraction of primary neurons by the cell mitochondria isolation kit (C3601, Beyotime) according to the manufacturer’s instructions. The primary neurons were incubated for 10 min in 0.1 ml ice-cold mitochondrial lysis buffer and homogenized 30 times. Then the nuclei were removed by centrifuging at 600 × g for 10 min at 4°C. We collected the cytosol fraction by centrifuging the supernatant at 11000 × g for 10 min at 4°C and the intact mitochondria were located in the precipitate. Finally, we isolated dsDNA extraction in cytosol by Animal Genomic DNA Quick Extraction Kit (D0065S, Beyotime) according to the manufacturer’s protocol.

### PCR

We evaluated cytoplasmic dsDNA (double stranded DNA) copy number by quantitative real-time PCR, as previously described [15]. The sequences of primers were as follows: Nd1 (forward-CTAGCAGAAACAAACCGGGC reverse-CCGGCTGCGTATTCTACGTT), ND4 (forward-AACGGATCCACAGCCGTA reverse-AGTCCTCGGGCCATGATT), 16 s (forward-CACTGCCTGCCCAGTGA reverse-ATACCGCGGCCGTTAAA), HK2 (forward-GCCAGCCTCTCCTGATTTTAGTGT reverse-GGGAACACAAAAGACCTCTTCTGG), and Tert (forward-CTAGCTCATGTGTCAAGACCCTCTT reverse-GCCAGCACGTTTCTCTCGTT).

### Statistical analysis

All results were reported as mean ± standard deviation (SD). The fluorescence intensity and western blot results were analyzed by ImageJ software. Statistical differences among the groups were analyzed using one-way ANOVA followed by Tukey post hoc test. The unpaired Student's t-test was used to compare differences between two groups using Prism software 6.04 (GraphPad Software, Inc.). Statistical significance was set at *p* < 0.05.

## Results

### H151, a specificity STING inhibitor, could alleviate neuroinflammation, reduce neuronal apoptosis, and relieve neurological dysfunction after SAH

**Fig. 1:**
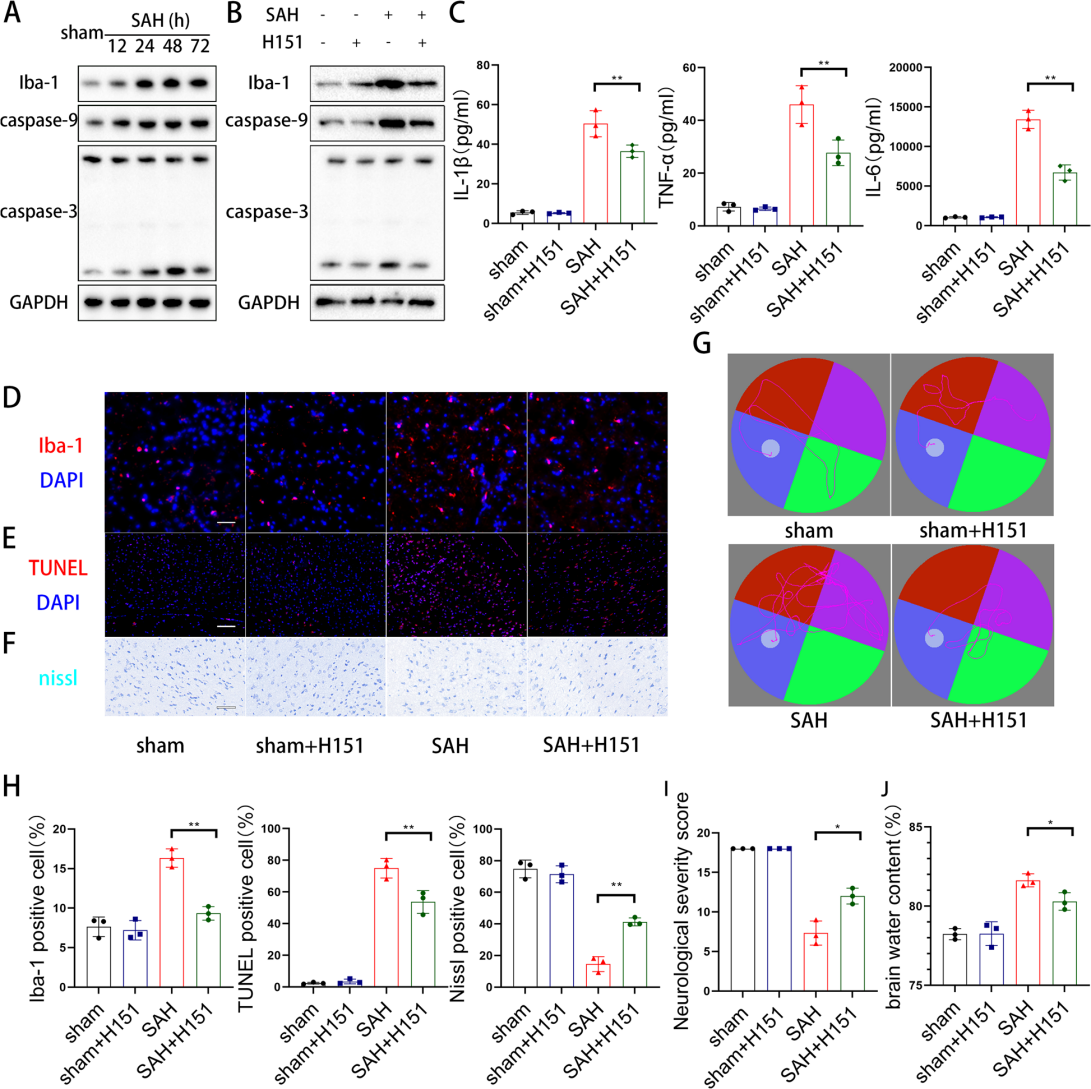
H151, a specificity STING inhibitor, could alleviate neuroinflammation, reduce neuronal apoptosis, and relieve neurological dysfunction after SAH. **A** Western blot analysis of Iba-1, caspase-3, caspase-9 expression. (*n* = 3 samples). **B** Western blot analysis of Iba-1, caspase-3, caspase-9 expression. (*n* = 3 samples). **C** Elisa analysis of IL-1β, TNF-α and IL-6. (*n* = 3 samples) (** *P* < 0.01). **D**, **H** Representative immunofluorescence images and quantitative analyses of Iba-1 in cerebral cortex. (** *P* < 0.01) (scale bar is 50μm). **E**, **H** Representative immunofluorescence images and quantitative analyses of TUNEL in cerebral cortex. (** *P* < 0.01) (scale bar is 200μm). **F**, **H** Representative images and quantitative analyses of nissl stain in cerebral cortex. (** *P* < 0.01) (scale bar is 50μm). **G** Representative swimming tracks of the mice in all four groups of the MWM task. **I**, **L** The neurological dysfunction were analyzed by neurological severity scores and brain water content after SAH. (* *P* < 0.05,** *P* < 0.01)

Given the important role of neuroinflammation and neuronal apoptosis in early brain injury after SAH, we tested the activation of microglia and cell apoptosis at different time points after SAH and the western blot analysis showed that the protein level increase of Iba-1, caspase-9, and cleaved caspase-3 increased after SAH, with peak at 48h after SAH (Fig. 1A). To further evaluate the effect of the cGAS-STING pathway on the outcome of SAH, we examined the level of neuroinflammation, cell apoptosis, and metabolism of neurons following H151 treatment. To begin with, we found that the increased protein levels of Iba-1, caspase-9, and cleaved caspase-3 were reversed by H151 treatment (Fig. 1B). In addition, the brain cortex inflammatory factor IL-1β, IL-6, and TNFα increased after SAH and significantly decreased with H151 treatment (Fig. 1C), meanwhile immunofluorescence results demonstrated that H151 reduced the activation of microglia and cell apoptosis, and improved neuronal metabolism (Fig. 1D-F, H). We examined the memory function and NSS score to detect the protective effects of H151 on neurological function. During the water maze test, H151-treated mice exhibited better learning capacity than the SAH group (Fig. 1G). Finally, H151 treatment enhanced neurological severity scores and reduced brain water content (Fig. 1I, J).

### cGAS and STING were up-regulated in neurons and astrocytes respectively after SAH

**Fig. 2:**
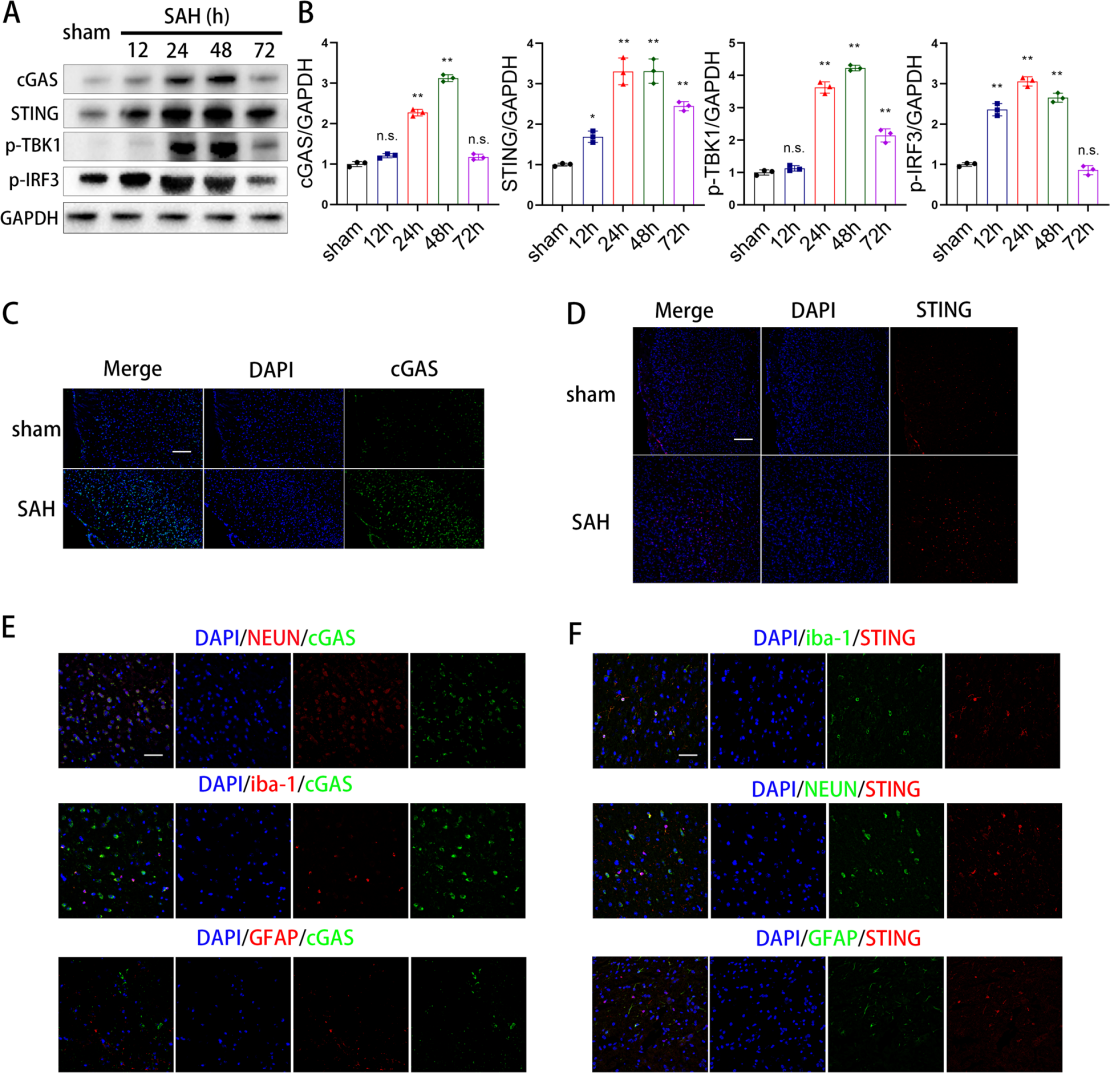
cGAS and STING were up-regulated in neurons and astrocytes respectively after SAH. A, **B** Western blot analysis and quantitative analyses of cGAS, STING, IRF3 and TBK1 expression. (*n* = 3 samples) (* *P* < 0.05,** *P* < 0.01). **C** Representative immunofluorescence images of cGAS in cerebral cortex. (scale bar is 200μm). **D** Representative immunofluorescence images of STING in cerebral cortex. (scale bar is 200μm). **E** Dual immunofluorescence staining of cGAS with NEUN, Iba-1 and GFAP. (scale bar is 50μm). **F** Dual immunofluorescence staining of STING with NEUN, Iba-1 and GFAP. (scale bar is 50μm)

In view of the anti-inflammatory function of H151, we tested the time course of cGAS, STING, p-IRF3, and p-TBK1 expression in SAH tissue (Fig. 2A), and western blot assessments demonstrated that their expression markedly increased and peaked at 24h or 48h after SAH (Fig. 2B). These results were confirmed using immunostaining (Fig. 2C, D). Further immunofluorescence showed that the cGAS were mostly expressed in neurons (labeled by NEUN) 48 h after SAH rather than astrocytes (labeled by GFAP) and microglia cells (labeled by iba-1) (Fig. 2E). However, STING was mostly expressed in microglial cells rather than in neurons and astrocytes (Fig. 2F).

### The cGAMP transferred from neurons to microglia activates the type I interferon response of microglia

**Fig. 3:**
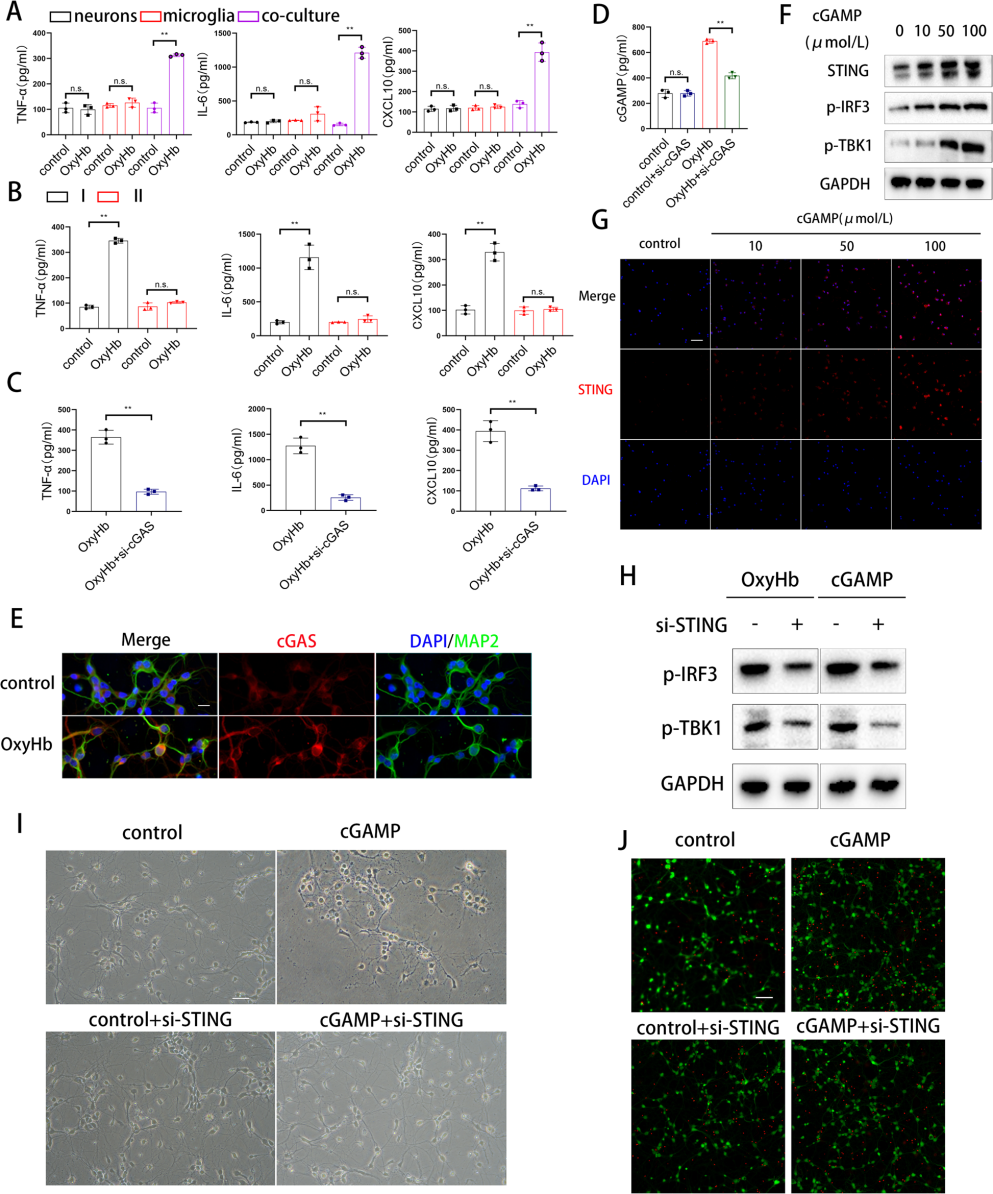
The cGAMP transferred from neurons to microglia activates the type I interferon response of microglia. **A**, **B**, **C** Elisa analysis of TNF-α, IL-6 and CXCL10. (** *P* < 0.01). **D** Elisa analysis of cGAMP. (** *P* < 0.01). **E** Dual immunofluorescence staining of cGAS with MAP2 in neurons. (scale bar is 10μm). **F** Western blot analysis of STING, IRF3 and TBK1 expression. **G** Representative immunofluorescence images of STING in microglia. (scale bar is 100μm). **H** Western blot analysis of IRF3 and TBK1 expression. **I** Representative photomicrographs of primary cortical neurons. (scale bar is 50μm). **J** Representative immunofluorescence images of PI (red) and Calcein (green) in primary cortical neurons. (scale bar is 50μm)

We further explored the activation mode of the cGAS-cGAMP-STING axis by co-culturing primary neurons and microglia. First, we administered OxyHb to neurons, microglia, and the co-culture system, and found that the type I interferon response was triggered only in the co-culture system (Fig. 3A). Subsequently, we examined whether type I interferon response was triggered in an indirect co-culture system. As shown in Fig. 3B, type I interferon response was triggered in microglia fed with OxyHb treated neuronal supernatant and reversed by neuron-si-cGAS (Fig. 3C). Combining the above results, we suspected that cGAMP plays a significant role in triggering type I interferon response. Immunofluorescence showed that cGAS was increased after OxyHb treatment (Fig. 3E), and ELISA results showed that the increased secretion of cGAMP by neurons after OxyHb administration was blocked by si-cGAS (Fig. 3D), meanwhile, STING, IRF3, and TBK1 were activated by cGAMP administration and increased with the concentration gradient (Fig. 3F, G). Furthermore, the activation of IRF3 and TBK1 could be attenuated by si-STING and H151 after cGAMP treatment (Fig. 3H). Moreover, we detected the condition of the neurons in the co-culture system, and the neurons displayed axonal shortening and higher mortality after cGAMP administration and si-STING ameliorated these changes (Fig. 3I and J).

### cGAMP was transferred by VRAC channels

**Fig. 4:**
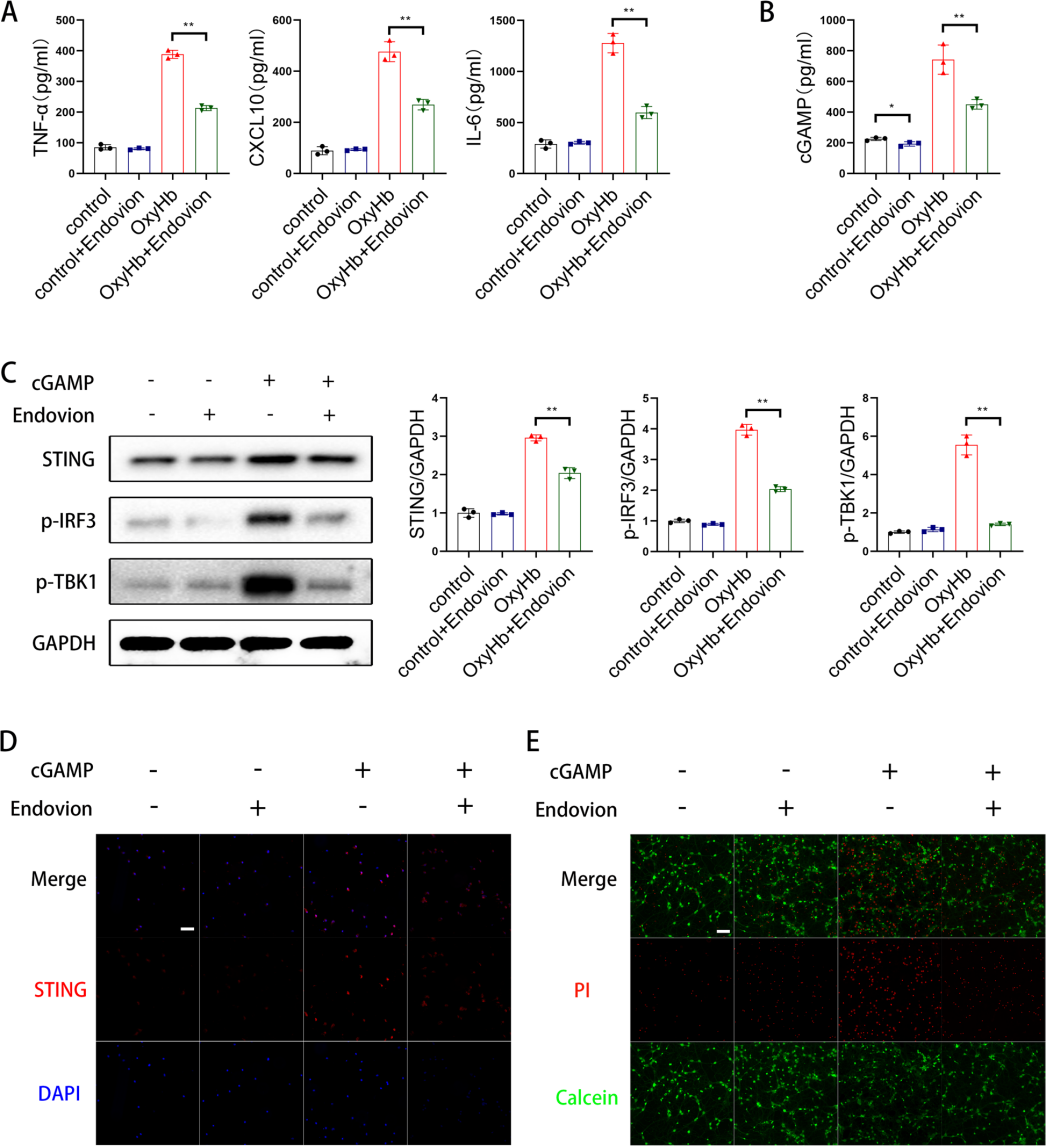
cGAMP was transferred by VRAC channels. **A** Elisa analysis of TNF-α, IL-6 and CXCL10. (** *P* < 0.01). **B** Elisa analysis of cGAMP. (** *P* < 0.01). **C** Western blot analysis of STING, p-IRF3 and p-TBK1 expression. **D** Representative immunofluorescence images of STING in microglia. (scale bar is 100μm). **E** Representative immunofluorescence images of PI (red) and Calcein (green) in primary cortical neurons. (scale bar is 50μm)

As reported in a previous study, cGAMP can be transferred from virus host cells to immune cell by the Lrrc8 transporter [16]. To determine the transfer mode of cGAMP from neurons to microglia, we blocked the Lrrc8/VRAC channels using endovion, and found that endovion decreased the activation of type I interferon response in the co-culture system (Fig. 4A) and the cGAMP content of the supernatant (Fig. 4B). In addition, endovion decreased the protein levels of STING, p-IRF3, and p-TBK1 (Fig. 4C, D) in microglia, and reduced neuronal apoptosis in the co-culture system (Fig. 4E).

### cGAS in neurons was primarily activated by intracytoplasmic mtDNA

**Fig. 5:**
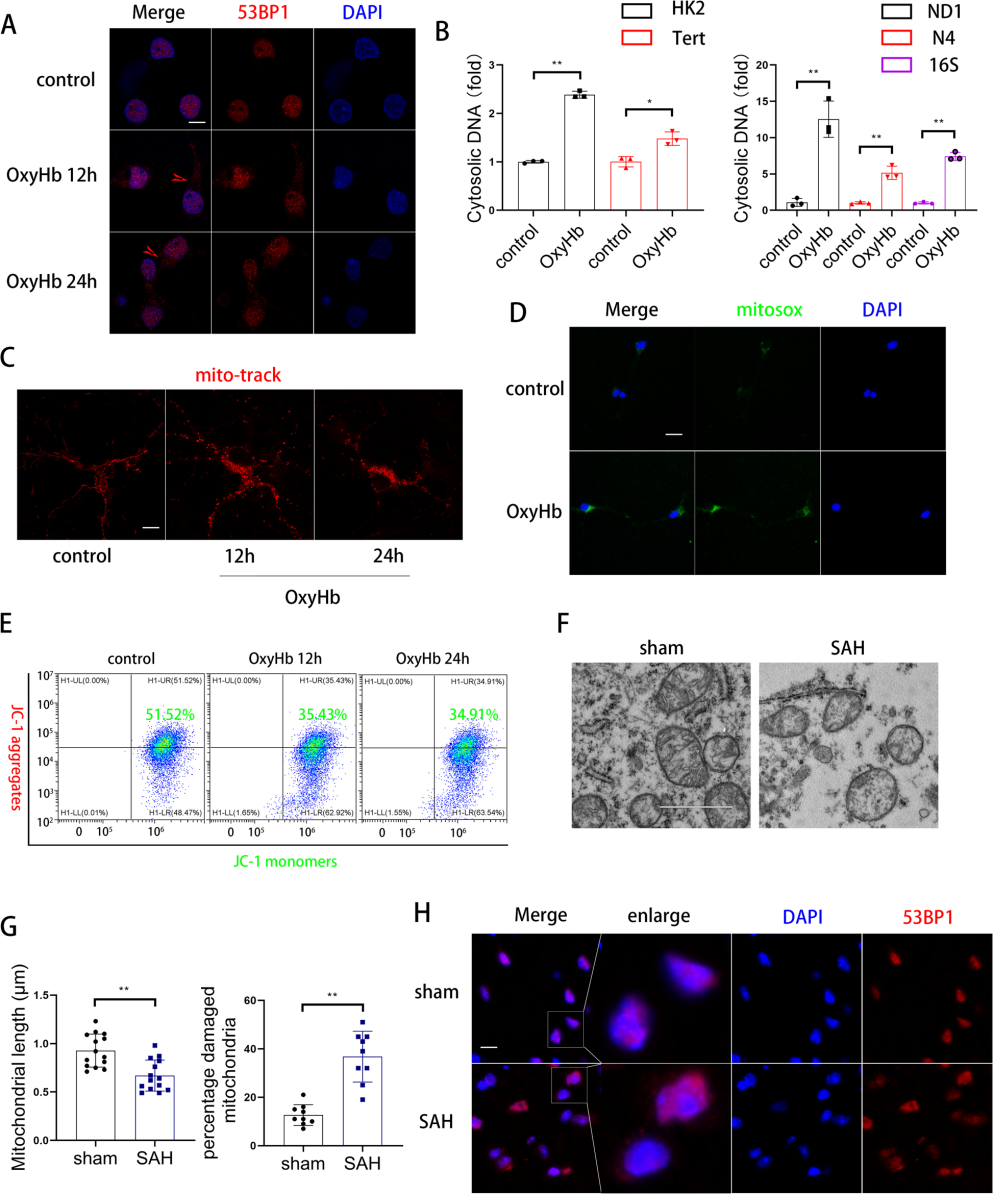
cGAS in neurons was primarily activated by intracytoplasmic mtDNA. **A** Representative immunofluorescence images of 53BP1 (red) in primary cortical neurons. (scale bar is 5μm). **B** QPCR analysis of cytosolic dsDNA. (* *P* < 0.05,** *P* < 0.01). **C** Representative immunofluorescence images of mito-track (red) in primary cortical neurons. (scale bar is 3μm). **D** Representative immunofluorescence images of mitosox (green) in primary cortical neurons. (scale bar is 20μm). **E** Flow cytometry analysis of JC-1 of primary cortical neurons. **F**, **G** Representative transmission electron microscope images and quantitative analyses in cerebral cortex. (scale bar is 2μm) (** *P* < 0.01). **H** Representative immunofluorescence images of 53BP1 (red) in cerebral cortex. (scale bar is 20μm)

To explore the cause of cGAS activation, we examined the distribution of 53BP1, which showed the location of the damaged DNA [17]. Immunofluorescence revealed that 53BP1 appeared in the cytoplasm after OxyHb treatment (Fig. 5A). We further assessed the source of dsDNA in the cytoplasm by qPCR and found that cytoplasmic mtDNA increased more than nuclear DNA after OxyHb treatment, indicating that cGAS was mostly activated by cytoplasmic mtDNA (Fig. 5B). We sought to determine mitochondrial dysfunction after OxyHb treatment. First, we investigated the mitochondrial membrane potential using MitoTrack and JC-1. As shown in Fig. 5C,E,F OxyHb treatment significantly altered mitochondrial morphology and decreased mitochondrial membrane potential. The OxyHb treatment improved the intensity of mitosox compared to the control group (Fig. 5D).

Next, we examined whether mitochondrial dysfunction occurs after SAH in vivo. The transmission electron microscop results suggested that the mitochondrial length was shortened, and the percentage of damaged mitochondria significantly increased after SAH (Fig. 5G). Meanwhile, immunofluorescence of 53BP1 demonstrated that mtDNA was damaged after SAH (Fig. 5H).

### Pharmacologically blocking mPTP ameliorated activation of the mtDNA-cGAS-STING axis

**Fig. 6:**
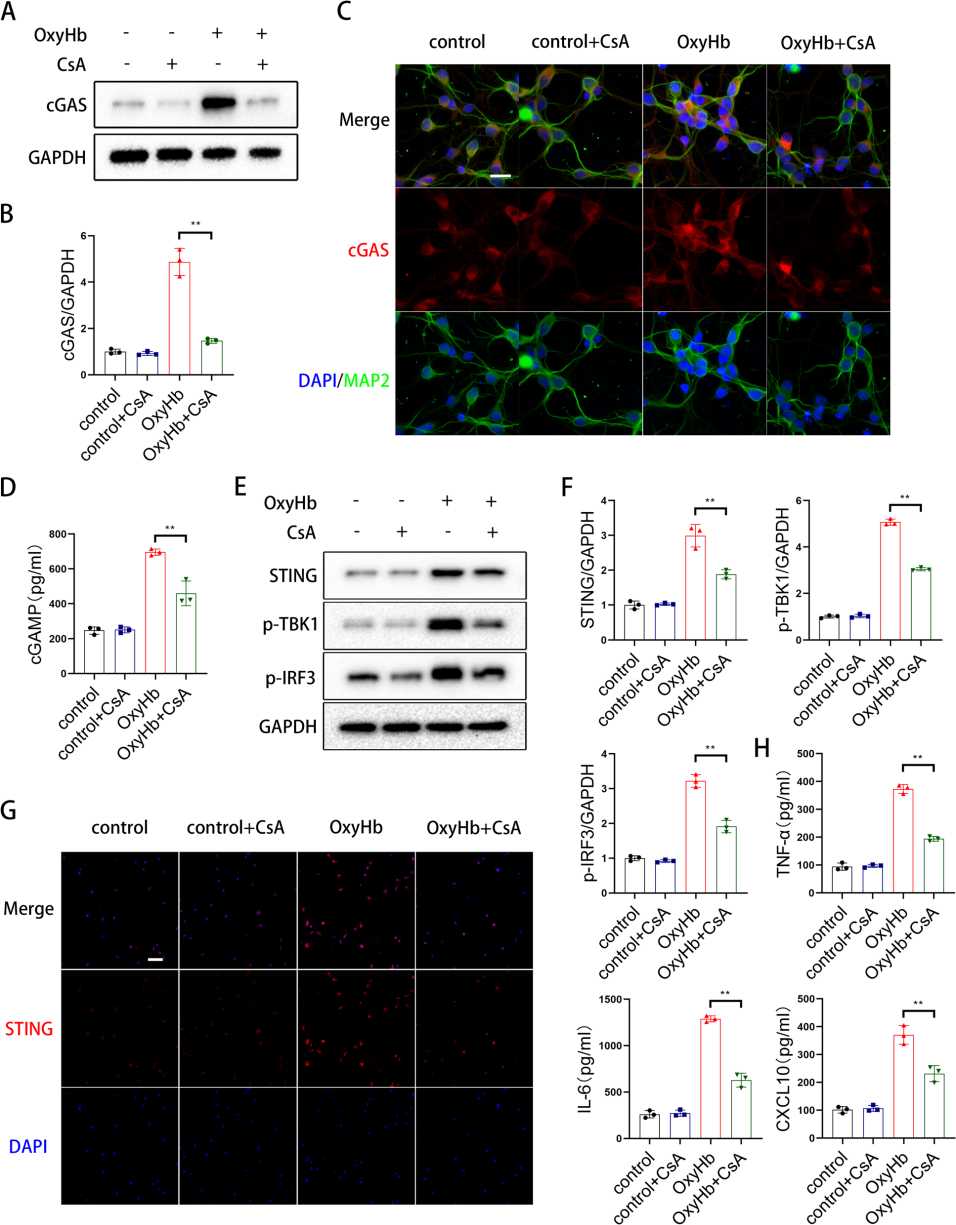
Pharmacologically blocking mPTP ameliorated activation of the mtDNA-cGAS-STING axis. **A**, **B** Western blot analysis of cGAS expression. **C** Dual immunofluorescence staining of cGAS with MAP2 in neurons. (scale bar is 10μm). **D** Elisa analysis of cGAMP. (** *P* < 0.01). **E**, **F** Western blot analysis of STING, p-IRF3 and p-TBK1 expression. **G** Representative immunofluorescence images of STING in microglia. (scale bar is 100μm). **H** Elisa analysis of TNF-α, IL-6 and CXCL10. (** *P* < 0.01)

As reported previously, mtDNA was transitted by mPTP (mitochondrial permeability transition pore) [13]. To determine whether blocking the mPTP could reduce the activation of the type I interferon response, we blocked the mPTP with CsA. The experimental results showed that CsA treatment reduced the protein level of cGAS (Fig. 6A-C) and the cGAMP content of the supernatant (Fig. 6D), meanwhile, CsA treatment decreased the protein levels of STING, p-IRF3, and p-TBK1 (Fig. 6E-G) in microglia and the type I interferon response (Fig. 6H).

### Inhibition of STING could reverse cGAMP-induced microglial M1 type polarization both in vitro and in vivo

**Fig. 7:**
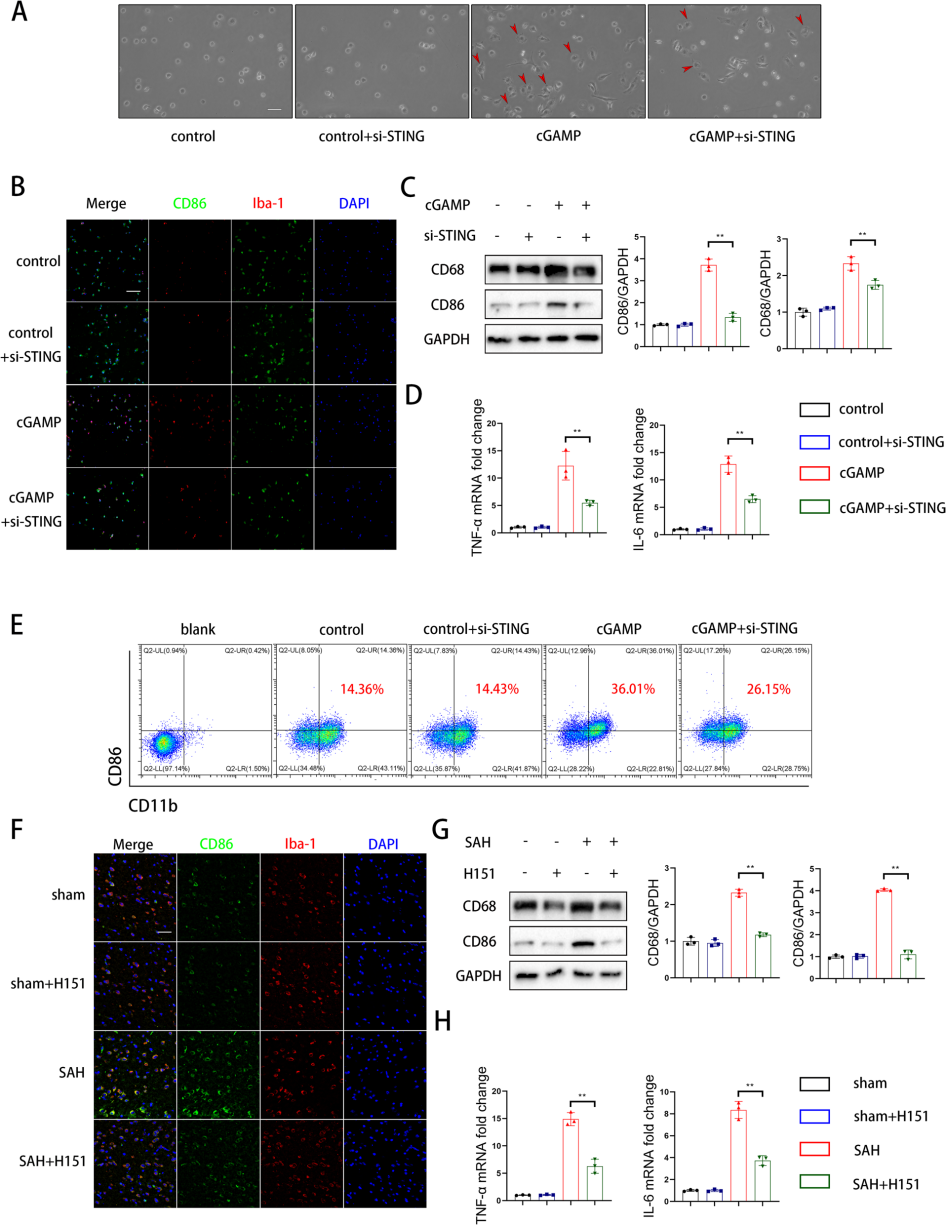
Inhibition of STING could reverse cGAMP-induced microglial M1 type polarization both in vitro and in vivo. **A** Representative photomicrographs of microglia. (scale bar is 50μm). **B** Dual immunofluorescence staining of CD86 with Iba-1 of microglia. (scale bar is 100μm). **C** Western blot analysis and quantitative analyses of CD86 and CD68 expression. (** *P* < 0.01). **D** QPCR analysis of IL-6 and TNF-α. (** *P* < 0.01). **E** Flow cytometry analysis of CD86 and CD11b of microglia. **F** Dual immunofluorescence staining of CD86 with Iba-1 in cerebral cortex. (scale bar is 100μm). **G** Western blot analysis and quantitative analyses of CD86 and CD68 expression. (** *P* < 0.01). **H** QPCR analysis of IL-6 and TNF-α. (** *P* < 0.01)

To explore the molecular and cellular mechanisms of microglia in the inflammatory response following SAH, we investigated the activation and polarization of microglia, both in vitro and in vivo. The experimental results showed that si-STING attenuated cGAMP-induced amoeba activation, suggesting that inhibition of STING could restrain microglial activation in vitro (Fig. 7A). We further assessed the polarization direction of microglia after cGAMP administration using immunofluorescence, western blot, and flow cytometry. Fluorescence showed that dual immunofluorescence labeling using CD86 and Iba-1 indicated that inhibition of STING could reverse cGAMP-induced microglial M1 type polarization (Fig. 7B). Western blot and flow cytometry showed that the expression of CD86 and CD68 were increased with cGAMP administration and decreased after STING inhibition (Fig. 7C and E). As illustrated in Fig. 7D, both TNF-α and IL-6 mRNA levels were increased following cGAMP treatment and were subsequently reversed with si-STING (Fig. 7D).

Next, we examined whether M1 type polarization occurred after SAH in vivo. Dual immunofluorescence labeling using CD86 and Iba-1 indicated that the microglia were polarized towards the M1 inflammatory type after SAH and reversed by H151 (Fig. 7F). At the same time, western blot results revealed that the expression of CD86 and CD68 increased after SAH and decreased with H151 administration (Fig. 7G). Notably, consistent with our in vitro findings, these mRNA levels exhibited a similar increase after SAH and were mitigated following H151 treatment (Fig. 7H).

### Neuroprotective effects of intracerebroventricular injection of AAV-cGAS and AAV-STING

**Fig. 8:**
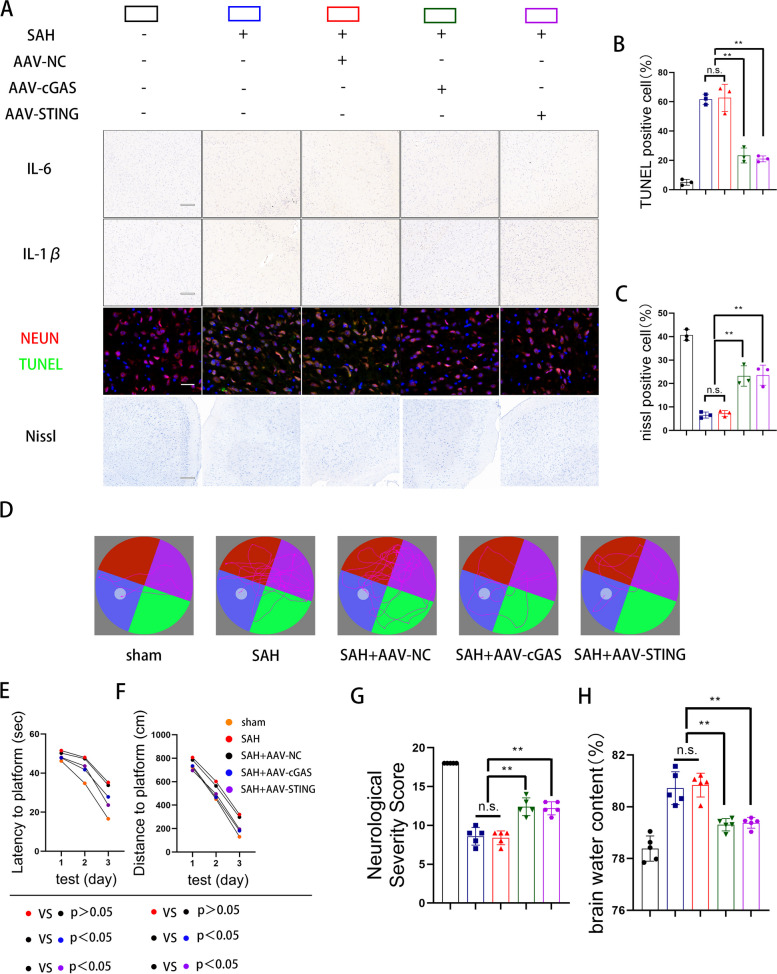
Neuroprotective effects of intracerebroventricular injection of AAV-cGAS and AAV-STING. **A** Representative immunohistochemical images of IL-6 and IL-1β in cerebral cortex. (scale bar is 300μm). **A**, **B** Representative dual immunofluorescence images and quantitative analyses of TUNEL and NEUN in cerebral cortex. (** *P* < 0.01) (scale bar is 50μm). **A**, **C** Representative images and quantitative analyses of nissl stain in cerebral cortex. (** *P* < 0.01) (scale bar is 300μm). **D**, **E**, **F** Representative swimming tracks of the mice in all five groups of the MWM task and quantitative analyses of latency and distance. **G**, **H** The neurological dysfunction were analyzed by brain water content and neurological severity scores after SAH. (* *P* < 0.05,** *P* < 0.01)

To examine the validity of the proposed mechanism in vivo, we performed AAV(adeno-associated virus)-neuron-cGAS and AAV-microglia-STING transfections 3 days before SAH. As expected, specific inhibition of cGAS in neurons and STING in microglia attenuated neuroinflammation, decreased neural apoptosis, ameliorated the existent state of neurons, and increased the activity of neurons (Fig. 8A, B, and C).

To detect the protective effects on neurological function of AAV-neuron-cGAS and AAV-microglia-STING, we examined memory function and NSS scores. During the water maze test, specific inhibition of cGAS of neurons and STING of microglial mice exhibited better learning capacity than the AAV-NC group, reflecting less time and distance before reaching the platform (Fig. 8D, E, F). Finally, AAV enhanced neurological severity scores and reduced brain water content (Fig. 8G, H).

## Discussion

Subarachnoid hemorrhage, especially that caused by ruptured intracranial aneurysms, may result in catastrophic consequences for patients, including diffuse cerebral edema and vasospasm [4, 18–20]. Although nimodipine remains the first-line treatment for vasospasm after SAH in clinical practice, little progress has been made in the development of drug-based therapies that can be used to inhibit early brain injury, which is a major prognostic factor. Reducing early brain injury, especially alleviating neuroinflammation, is critical for improving prognosis after SAH. The current study identified a neuroprotective role by inhibiting the I-type interferon reaction caused by the cGAS-cGAMP-STING signaling axis. Here, we demonstrated that neuron-derived cGAMP promotes microglial polarization to M1 type and triggers excessive neuroinflammation.

cGAS, the synthase for the second messenger cyclic GMP-AMP, can detect exogenous and endogenous double-stranded DNA after bacterial and viral infections. The cGAS-STING pathway is involved in several conditions, such as acute kidney injury, Parkinson’s disease, and ischemic stroke [11–13, 15, 21]. Excessive activation of the cGAS-cGAMP-STING signaling axis can trigger I-type interferon response and elicit injurious inflammation. Under stress conditions, nuclear and mitochondrial dsDNA leak into the cytoplasm and are recognized by cGAS. Subsequently, STING translocates from the endoplasmic reticulum through the endoplasmic reticulum-Golgi intermediate compartment to the Golgi, which signals downstream activation [15, 16].

Previous studies revealed that nuclear and mitochondrial dsDNA can activate the cGAS-cGAMP-STING signaling pathway. According to previous studies, reducing cGAS activation and microenvironmental inflammation by inhibiting mtDNA cytosolic leakage is effective [10, 11, 13]. We previously reported that mitochondrial dysfunction is a severe pathological state after SAH. Thus, we speculated that abnormal intracytoplasmic mtDNA is the initiating factor of the cGAS-cGAMP-STING signaling axis. To test this hypothesis, we identified the dsDNA origin by PCR, and the results showed that intracytoplasmic mtDNA fold increase was much greater than that of nuclear DNA. As Muhammad et al. reported, the systemic mtDNA levels were elevated after SAH and associated with systemic inflammatory response [22]. Meanwhile, the elevation of mtDNA after SAH was related to gender, age and complications. Differ to serum mtDNA, the cytosolic mtDNA of neurons were elevated more rapidly, causing an earlier neuroinflammatory response.

Microglia are resident, moving immune cell in the central nervous system microenvironment. Microglial assembly and phagocytosis are barriers to maintaining the stability of the central nervous system under physiological conditions [23, 24]. However, excessive microglial activation and neuroinflammation can cause neural apoptosis and nervous system dysfunction. Increasing evidence has shown that the cGAS-cGAMP-STING signaling axis plays a key role in numerous stroke diseases, such as ischemic stroke and sinus thrombosis. Previous studies showed that microglial activation and polarization are central to neuroinflammation and its aggravation. Recently, Li et al. reported that in ischemic stroke, inhibition of cGAS and microglial activation attenuated neuroinflammation, decreased neuronal apoptosis, and improved neural function in mice [25]. Kong et al. demonstrated that Inhibition of STING may serve as a potential therapeutic strategy to mitigate neuroinflammation and reduce ischemia reperfusion injury after ischemic stroke [26]. It has been reported that ischemia reperfusion injury induces mitochondrial dysfunction and leakage of mtDNA into the cytoplasm, eventually leading to M1 polarization of microglia. Blockade of STING promoted M1-type polarization to M2-type polarization through IFR3 and NF-κB, thereby mitigating neuroinflammation. To confirm the origin of neuroinflammation after SAH, we tested the polarization state of microglia, both in vitro and in vivo, and found that protein expression of CD86 and iNOS increased after SAH and cGAMP administration in microglia, and knockdown of cGAS in neurons and STING in microglia reversed the M1-type polarization of microglia.

Interestingly, multiple studies have shown the transfer of cGAMP between parenchyma and immune cell [16, 27–29]. Zhou et al. reported that cell-to-cell transmission of cGAMP is central to effective antiviral immunity via LRRC8/VRAC channels [16]. Another study showed that macrophages can take up extracellular cGAMP to engage in STING and amplify inflammatory response through connexin [30]. Cell-to-cell communication exist extensively under various pathological conditions.

As expected, our in vivo and in vitro experiments showed that the protein levels of cGAS and STING increased in the cerebral cortex after SAH. However, we detected that the expression of STING and I-type interferon responses were activated in microglia rather than in neurons. Therefore, we attempted to determine the role of cGAMP in microglial activation and polarization. Initially, we found that cGAMP concentration increased after SAH in vitro. We proved that both exogenous and neuron-derived cGAMP could promote the expression of STING and I-type interferon responses in microglia. Furthermore, elevated cGAMP levels can be reversed by cGAS knockdown, and the activation and polarization of microglia can also be interrupted by STING knockdown.

### Limitation

This study had several limitations. First, our subarachnoid hemorrhage model was induced using oxyhemoglobin. Although this is a widely used model, the physiological and pathological processes may differ from those of subarachnoid hemorrhage. Second, although the treatment of AAV vector gene therapy for aromatic l-amino acid decarboxylase deficiency has been proven valid, the application of AAV in humans with subarachnoid hemorrhage is difficult to predict. Third, the cGAS-cGAMP-STING signaling axis is involved in both acute and chronic pancreatitis. It would be important to explore the effect of cGAS-cGAMP-STING signaling axis after EBI.

## Conclusion

The cGAS-cGAMP-STING signaling axis is closely associated with neuroinflammation after subarachnoid hemorrhage. The transmission of cGAMP from neurons to microglia is a key molecular event that triggers type I interferon response, amplifies neuroinflammation, and leads to neuronal apoptosis. Understanding the physiological and pathological processes of type I interferon response may provide new diagnostic and treatment methods for regulating the survival and death of neuronal cells after SAH. Targeting cGAS triggered by cytoplasmic mtDNA may be useful for comprehensive clinical treatment of patients after SAH. Further studies targeting cGAS-specific antagonists for treating SAH are warranted.

### Supplementary Information

**Additional file 1. ** Supplementary Figure 1.

## Data Availability

The data used to support the fndings of this study are available from the corresponding author upon request.

## Abbreviations

SAH: Subarachnoid hemorrhage
EBI: Early brain injury
cGAMP: Cyclic GMP-AMP
mtDNA: Mitochondrial DNA
cGAS: Synthase for the second messenger cyclic GMP-AMP
STING: Cyclic GMP-AMP receptor stimulator of interferon genes
DCI: Delayed cerebral ischemia
SPF: Specified Pathogen Free
FBS: Fetal bovine serum
CsA: Cyclosporine
PVDF: Polyvinylidene Fluoride
MWM: Morris water maze
dsDNA: Double stranded DNA
mPTP: Mitochondrial Permeability Transition Pore
AAV: Adeno-Associated Virus
